# Systematic review and meta-analysis of pain management after tonsillectomy

**DOI:** 10.1038/s41598-024-85008-5

**Published:** 2025-01-09

**Authors:** Katharina Geißler, Daniel Scham, Winfried Meißner, Peter Schlattmann, Orlando Guntinas-Lichius

**Affiliations:** 1 Department of Otorhinolaryngology, Jena University Hospital, Am Klinikum 1, 07747 Jena, Germany; 2 Department of Anaesthesiology and Intensive Care Medicine, Jena University Hospital, Jena, Germany; 3 Institute of Medical Statistics, Informatics and Data Science, Jena University Hospital, Jena, Germany

**Keywords:** Tonsillectomy, Pain therapy, NSAID, Meta-analysis, Clinical trial, Diseases, Outcomes research

## Abstract

Tonsillectomy is one of the most common operations. Tonsillectomy is also one of the most painful surgical procedures. However, there is still no satisfactory standard for postoperative pain management. Four databases (Cochrane Library, Ovid Technologies, PubMed, Web of Science) were searched for the period from 1908 to 2019. The systematic literature review followed the Preferred Reporting Items for Systematic Reviews and Meta-Analyses (PRISMA) guidelines. Data were pooled using random-effects and fixed-effects models. Randomized controlled trials, reviews and meta-analyses were included. Primary outcomes were quantitative pain intensity in the first 24 h after tonsillectomy and on days 1, 3, and 7 postoperatively. The search yielded 1594 publications, of which 111 publications with 7566 patients, both children and adults, could be included. Intraoperative medication with intravenous dexamethasone significantly reduced pain (mean difference [MD] -0.42; 95% confidence interval [CI]: -0.61- -0.24). Among the local anesthetics, only the preoperative injection of levobupivacaine into the tonsillar compartment was able to provide sufficient pain reduction up to three days after tonsillectomy (MD: -1.92; 95% CI: -2.73 - -1.11). Preoperative or intraoperative administration of non-steroidal anti-inflammatory drugs (NSAIDs) significantly reduced pain (MD: -0.75; 95% CI: -0.87- -0.63). Steroids and NSAIDs are an important part of pain management after tonsillectomy.

## Introduction

Tonsillectomy, the surgical removal of the palatine tonsils, remains one of the most common surgical procedures in children and adults. For example, 36,155 tonsillectomies were performed in Germany in 2021^1^ and more than 30,000 tonsillectomies are performed annually in the United Kingdom^2^. Tonsillectomy is associated with severe postoperative pain lasting for many days^3^. A prospective cohort study taking part in the Quality Improvement in Postoperative Pain Treatment (QUIPS) registry has shown that tonsillectomy is one of the most painful surgical procedures even compared to major surgery procedures^4^. Despite of this, postoperative pain management is still not standardized and many patients are undertreated^5–7^.

Pain management after tonsillectomy typically uses a non-opioid drug for basic analgesia, combined with an opioid if needed, such as a non-steroidal anti-inflammatory drug (NSAID) including metamizole and. more recently, cyclooxygenase (COX)-2-inhibitors combined with an opioid such as morphine or tramadol. However, analgesic regimes vary widely between institutions and countries.

The current meta-analysis was performed to summarize the scientific evidence on postoperative pain management after tonsillectomy.

## Results

### Results of literature search

**Fig. 1:**
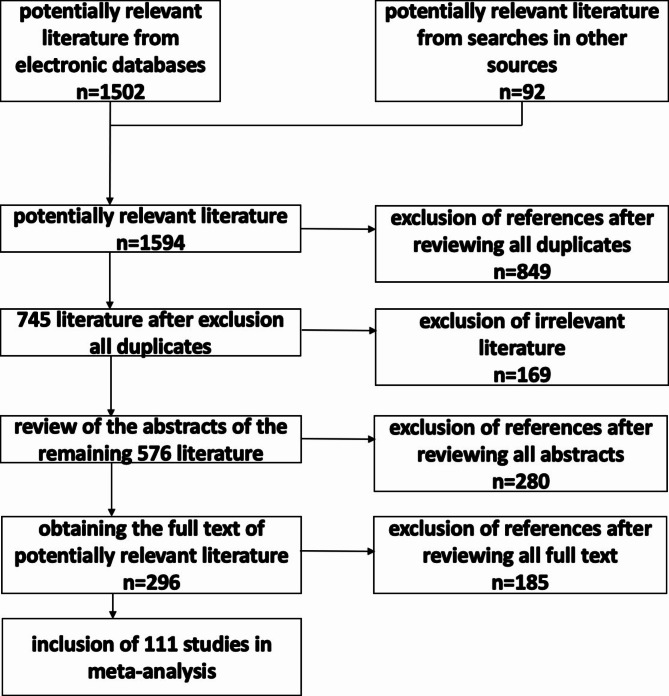
PRISMA flowchart.

The systematic literature search identified a total of 1502 publications in the four databases mentioned. In addition to the systematic literature search, a manual search identified 92 additional potentially relevant references. After reviewing all 1594 references, 849 duplicates were excluded. Based on the title, 576 of the remaining 745 publications could be included. Analysis of the abstracts led to the exclusion of 280 studies. From the full text, 111 of the remaining 296 publications were included (Fig. 1).

### Included studies

The 111 included trails included studies that analyzed the effects of drugs and/or other interventions. Most of the studies were of nonsteroidal anti-inflammatory drugs, steroids and local anesthetics. The other studies could be divided into studies analyzing the effects of antibiotics, opioids, non-opioid analgesics, coxibs, surgical techniques and various other therapies.

For the present meta-analysis, only the results of trials that included nonsteroidal anti-inflammatory drugs (*n* = 1382, age range 3 to 70 years), steroids (*n* = 907, age range 6 to 70 years) and local anesthetics (*n* = 2773, age range 3 to 86 years) are presented with respect to the following outcome measures: pain scales, primary and secondary bleeding and postoperative nausea and vomiting (PONV).

### Non-steroidal anti-inflammatory drugs

**Fig. 2:**
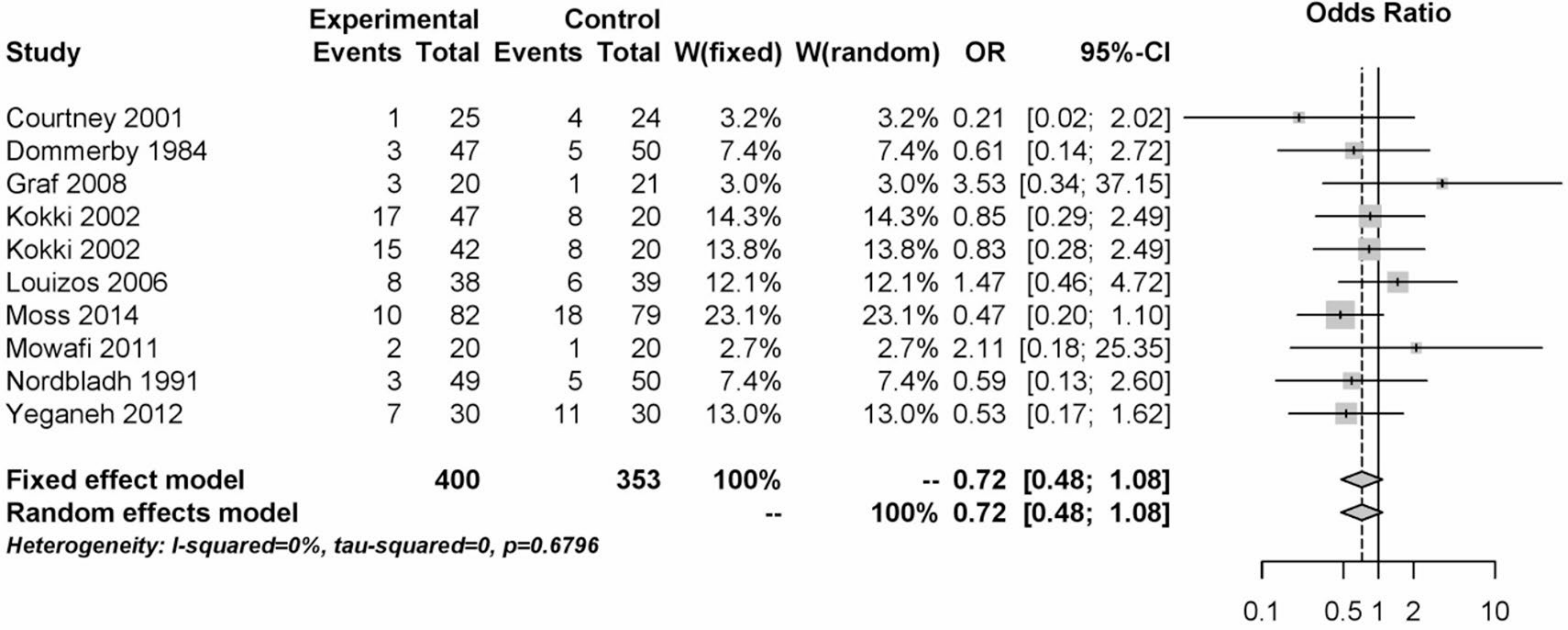
Forest plot for meta-analysis related to the outcome measure postoperative nausea and vomiting for the use of non-steroidal anti-inflammatory drugs.

A total of 18 studies could be assigned to the group of NSAIDs^7–24^. The PONV analysis shown in Fig. 2 included 10 studies with 753 study participants. There were no statistically significant differences between the fixed effects model (OR: 0.72; 95% CI: 0.48–1.08) and the random effects model (OR: 0.72; 95% CI: 0.48–1.08) concerning NSAID as experimental group and opioids, coxibs and other non-opioid analgetics such as naproxen, gabapentin or placebo as control group. No statistical heterogeneity was detected (I² = 0%).

**Fig. 3:**
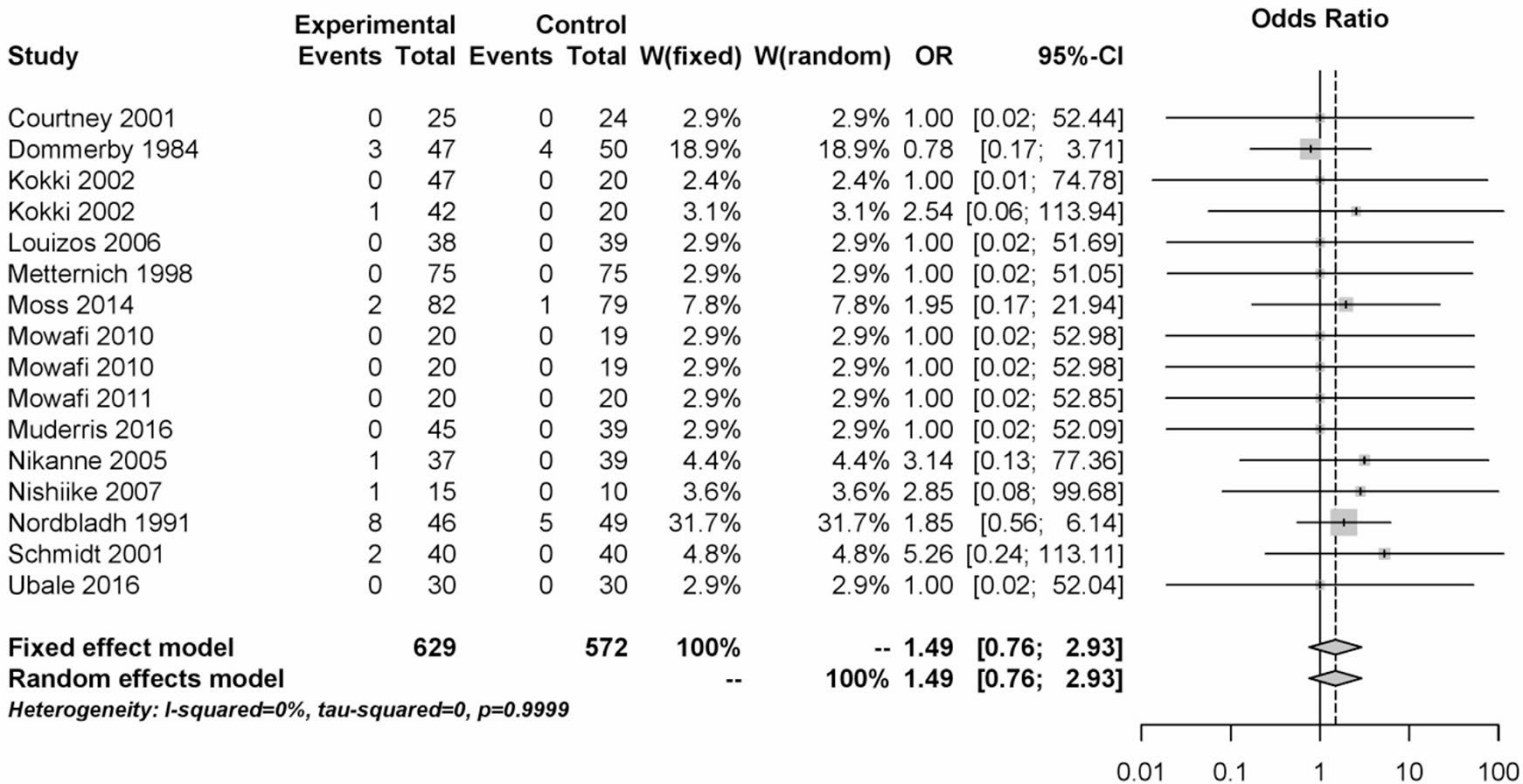
Forest plot for meta-analysis related to the outcome measure primary postoperative bleeding for the use of non-steroidal anti-inflammatory drugs.

The analysis of primary bleeding events is shown in Fig. 3. 16 studies with a total of 1201 patients were analyzed. There was no statistically significant difference between the NSAID as experimental and opioids, coxibs, naproxen or placebo as control groups (OR: 1.49; 95% CI: 0.76–2.93). No statistical heterogeneity was detected (I² = 0%).

**Fig. 4:**
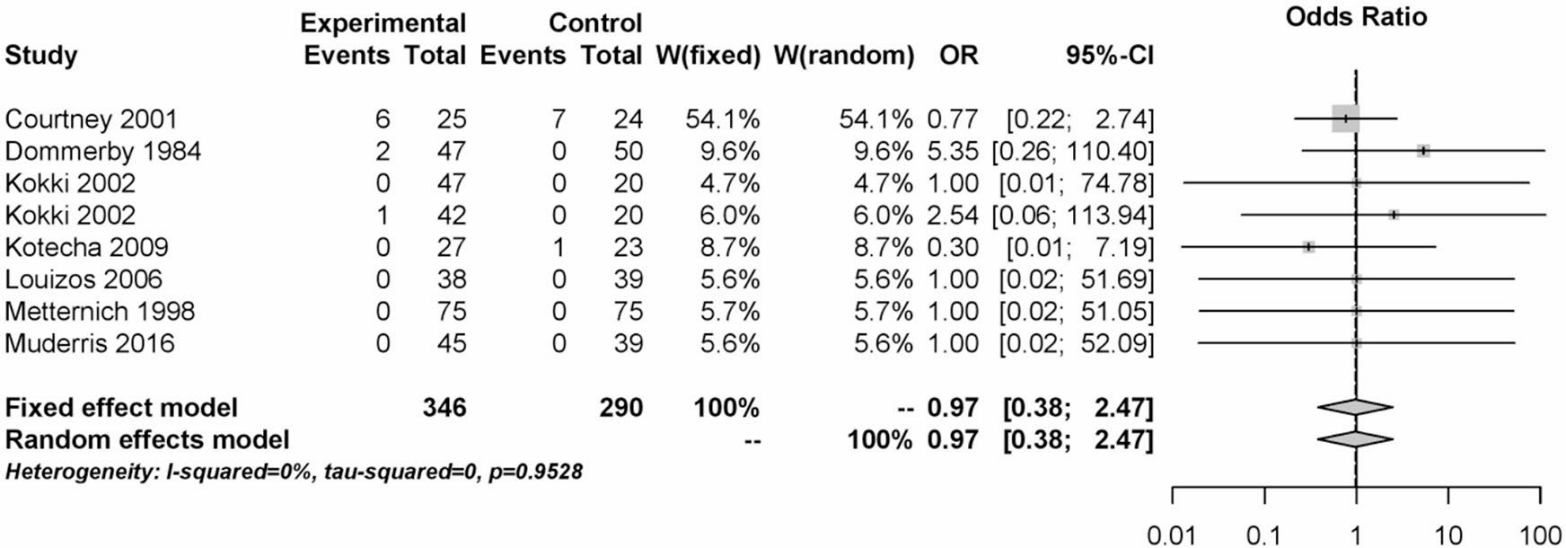
Forest plot for meta-analysis related to the outcome secondary postoperative bleeding for the use of non-steroidal anti-inflammatory drugs.

Similar to primary postoperative bleeding, there was also no significant difference in secondary postoperative bleeding between NSAID and opioids, coxibs, naproxen or placebo (OR: 0.97; 95% CI: 0.38–2.47). No statistical heterogeneity (I² = 0%) was detected among the 636 study participants from 8 different studies. The results are shown in Fig. 4.

**Fig. 5:**
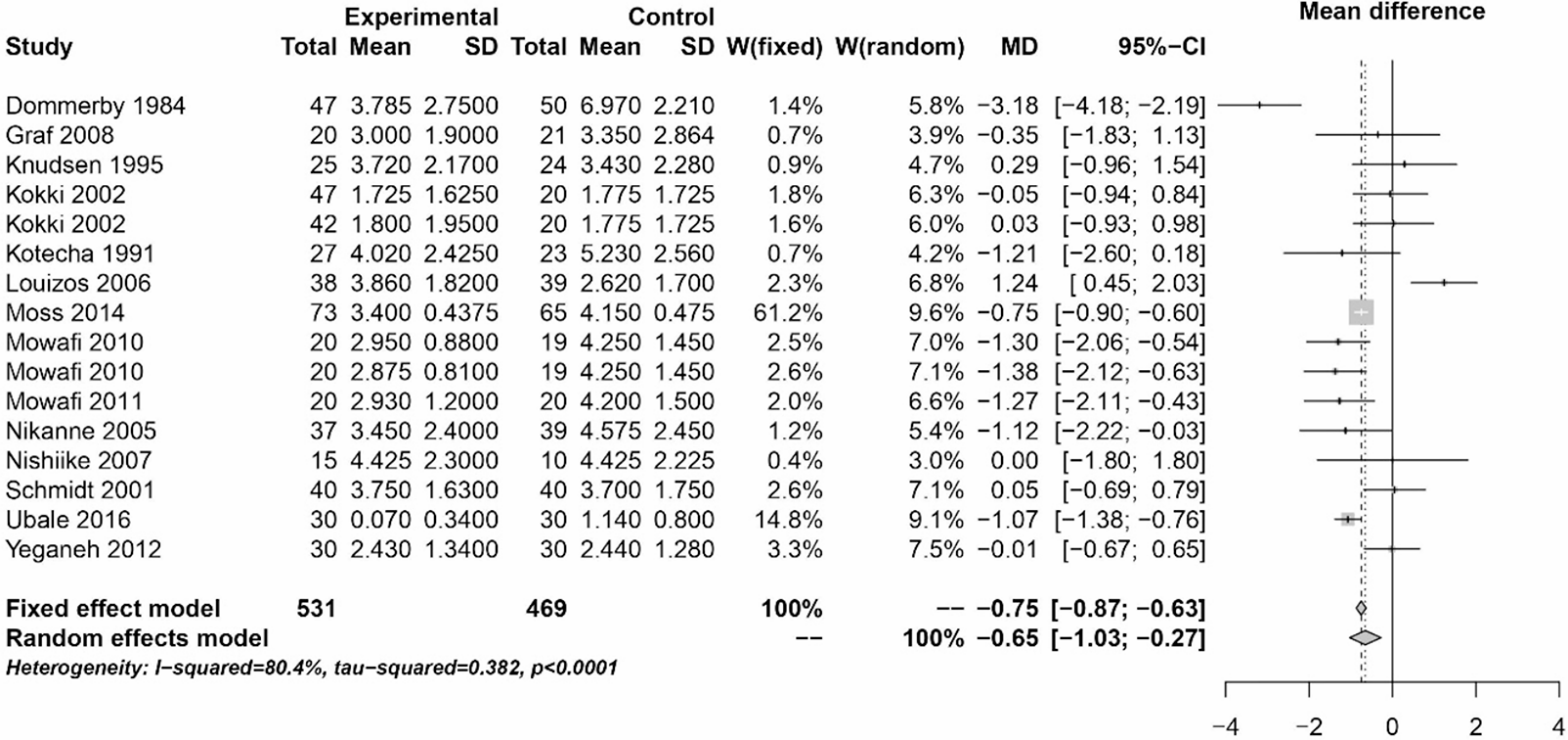
Forest plot for meta-analysis of pain measurement within the first 24 h for the use of non-steroidal anti-inflammatory drugs.

In the first 24 h pain analysis, 16 studies with 1000 patients could be analyzed. The fixed effects model (MD: -0.75; 95% CI: -0.87-0.63) and the random effects model (MD: -0.65; 95% CI: -1.03-0.27) showed significant pain relief in favor of NSAIDs (I² = 80.4%) compared to opioids, coxibs, naproxen or placebo. This is shown in Fig. 5.

**Fig. 6:**
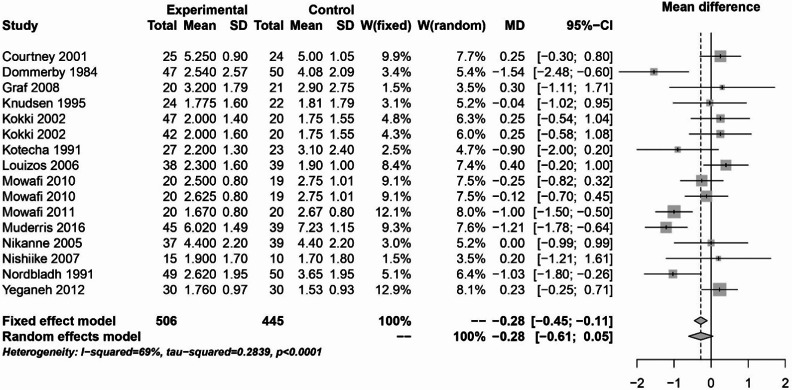
Forest plot for meta-analysis of pain measurement on first postoperative day for the use of non-steroidal anti-inflammatory drugs.

With regard to pain on the first postoperative day (Figs. 6), 16 studies with 951 patients were examined. A statistically significant difference between NSAIDs and opioids, coxibs, naproxen or placebo was found with the fixed effects model (MD: -0.28; 95% CI: -0.45 - -0.11). With the random effects model, however, the results were not significant (MD: -0.28; 95% CI: -0.61–0.05). The statistical heterogeneity was substantial (I² = 69%).

**Fig. 7:**
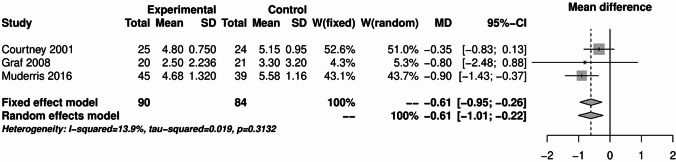
Forest plot for the meta-analysis of pain measurement on the third postoperative day for the use of non-steroidal anti-inflammatory drugs.

**Fig. 8:**
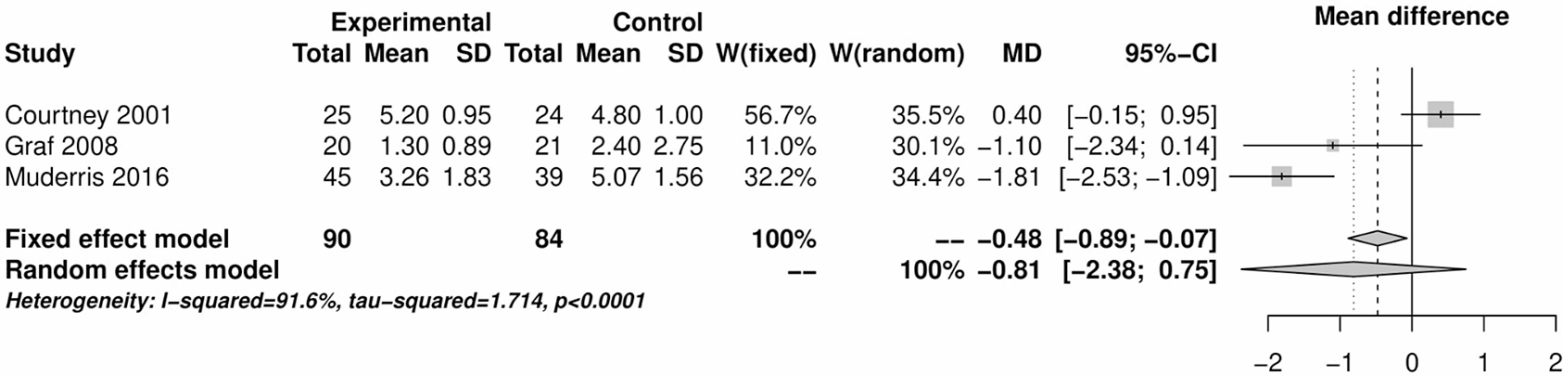
Forest plot for meta-analysis of pain measurement on day 7 postoperatively for the use of non-steroidal anti-inflammatory drugs.

Three studies with 174 patients each were analyzed for pain on postoperative day 3 and postoperative day 7. The results are shown in Figs. 7 and 8. On the third postoperative day, the NSAIDs were able to significantly reduce the pain compared to tramadol, naproxen or placebo. The pooled mean difference was − 0.61 for both models. The 95% confidence intervals were respectively − 0.95 - -0.26 for the fixed effects model and − 1.01 - -0.22 for the random effects model. There was some statistical heterogeneity among the included studies (I² = 13.9%).

There was considerable heterogeneity at postoperative day 7. The fixed effects model observed a reduction in pain for use of NSAID compared to tramadol, naproxen or placebo (MD: -0.48; 95% CI: -0.89-0.07), while the random effects model did not (MD: -0.81; 95% CI: -2.38-0.75).

### Steroids

**Fig. 9:**
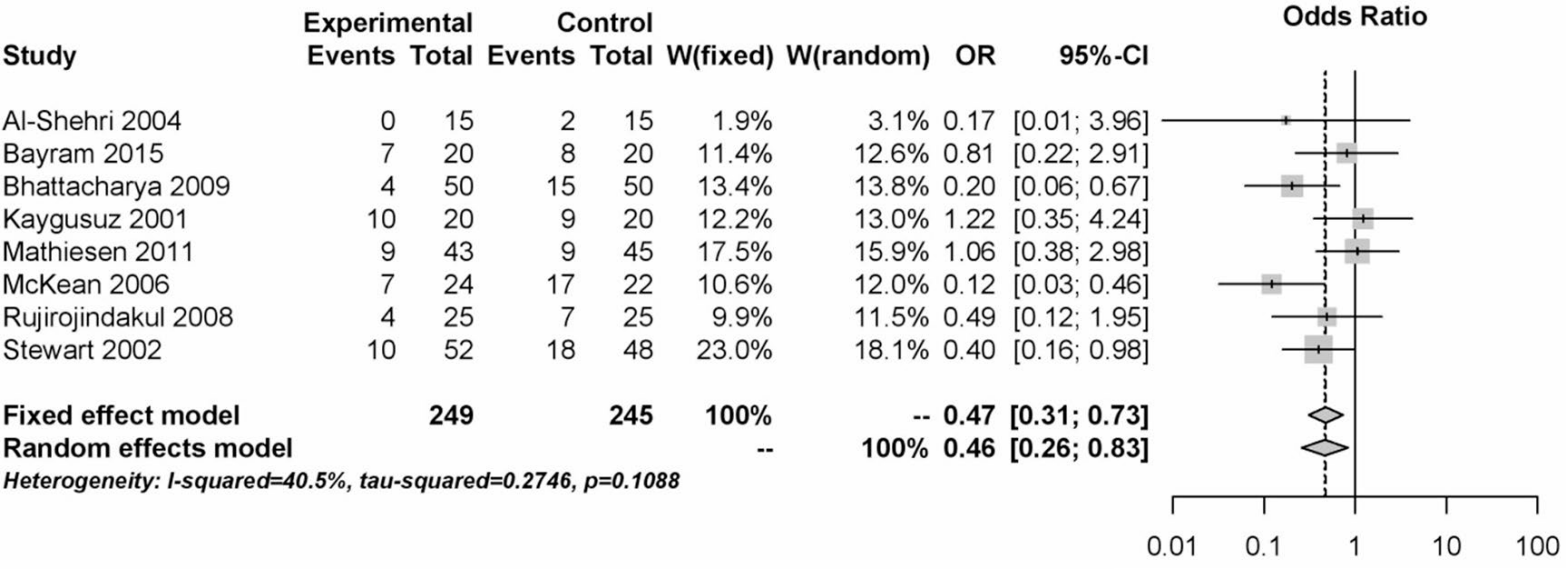
Forest plot for meta-analysis of postoperative nausea and vomiting for the use of steroids.

For steroids 15 literature references were found^25–39^. The PONV analysis is shown in Fig. 9, which showed a significant benefit of the steroids over placebo with low heterogeneity (I² = 40.5%). The OR was 0.47 (95% CI: 0.31–0.73) for the fixed effects model and 0.46 (95% CI: 0.26–0.83) for the random effects model in 494 participants studied.

**Fig. 10:**
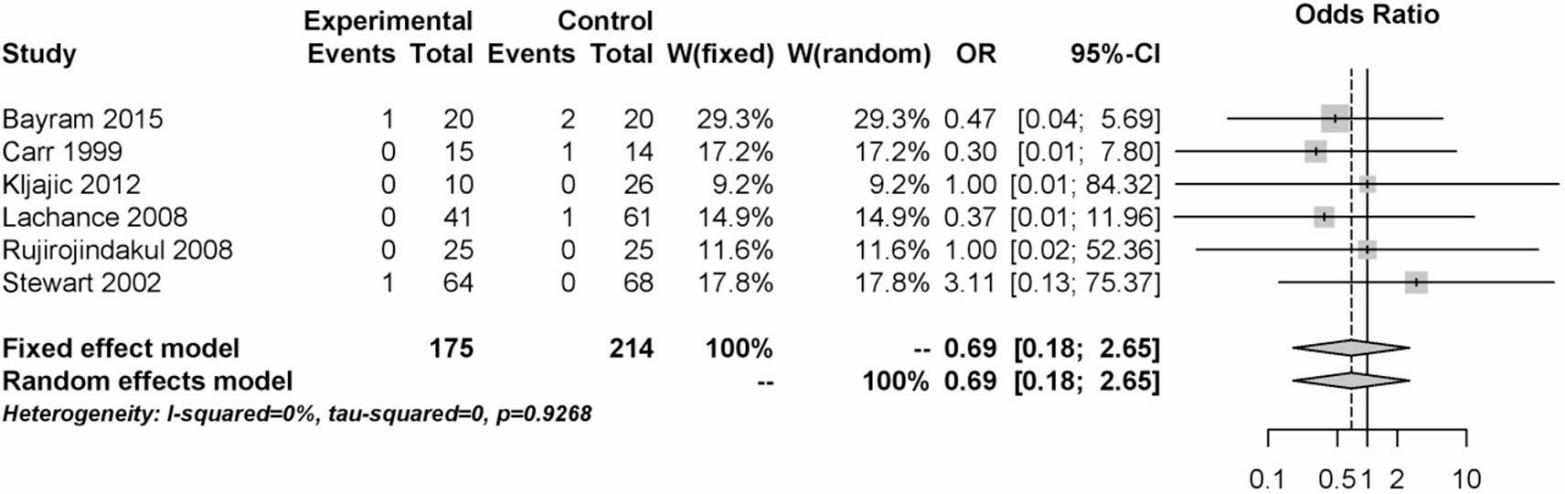
Forest plot for the meta-analysis of primary postoperative bleeding for the use of steroids.

For primary postoperative bleeding, there was no difference between the 175 patients in the steroid group and the 214 patients in the placebo group (OR: 0.69; 95% CI: 0.18–2.65, Fig. 10).

**Fig. 11:**
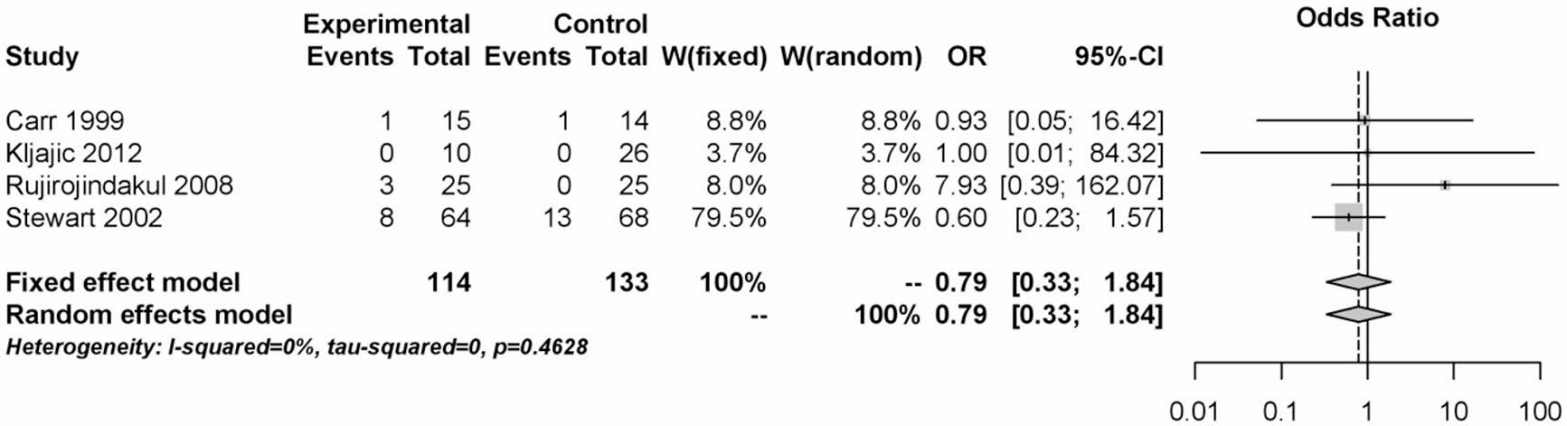
Forest plot for meta-analysis of secondary postoperative bleeding for the use of steroids.

In the case of secondary postoperative bleeding, 4 studies with a total of 247 patients were analyzed. As with primary postoperative bleeding, there was no significant difference between steroids and placebo (OR: 0.79; 95% CI: 0.33–1.84). Both postoperative bleeding analyses showed no heterogeneity (I² = 0%, Fig. 11).

**Fig. 12:**
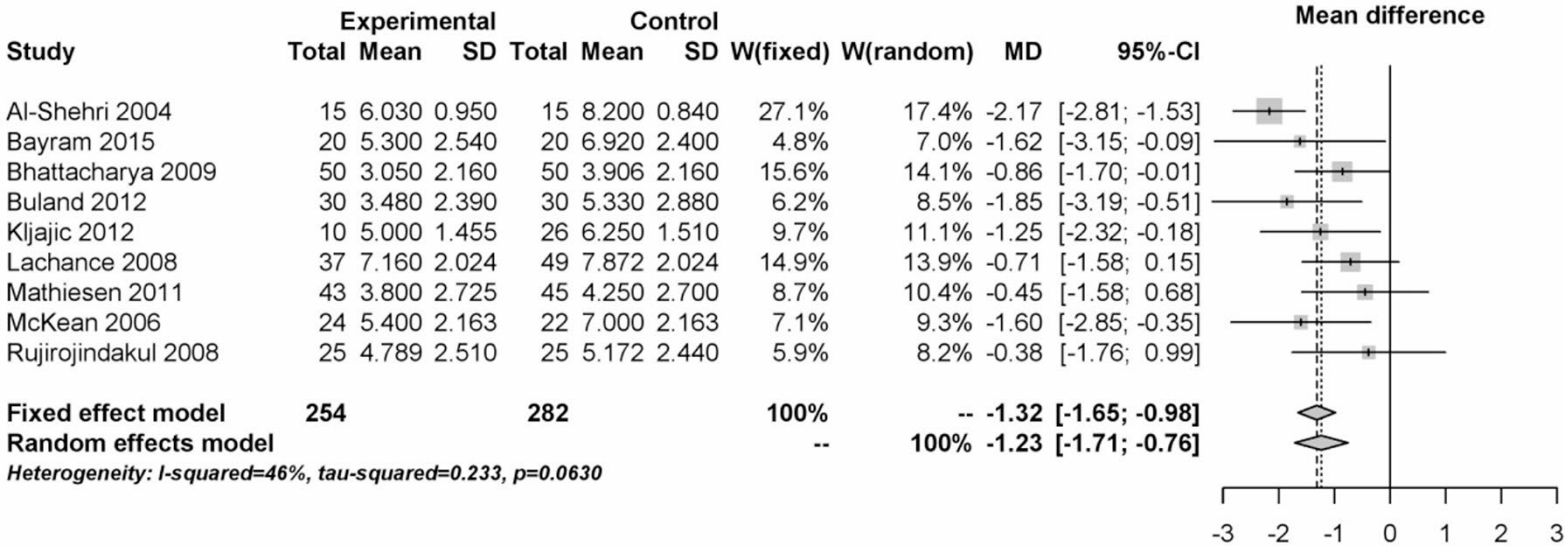
Forest plot for meta-analysis of pain measurement within the first 24 h for the use of steroids.

In the first 24 h postoperatively after tonsillectomy, steroids contributed significantly to pain reduction compared to placebo. This is shown in Fig. 12. In the 536 patients from 9 studies, a mean difference of -1.32 (95% CI: -1.65 - -0.98) was observed for the fixed effects model and − 1.23 (95% CI: -1.71 - -0.76) for the random effects model with moderate heterogeneity (I² = 46%).

**Fig. 13:**
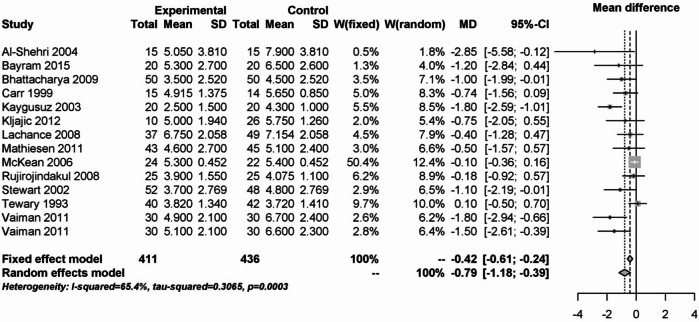
Forest plot for meta-analysis of pain measurement on day 1 postoperatively for the use of steroids.

The benefit of the steroids was also observed on the first postoperative day. However, there was substantial heterogeneity (I² = 65.4%). While the mean difference was − 0.42 (95% CI: -0.61- -0.24) for the fixed effects model, it was − 0.79 (95% CI: -1.18 - -0.39) for the random effects model. 847 patients from 14 studies were analyzed (Fig. 13).

**Fig. 14:**
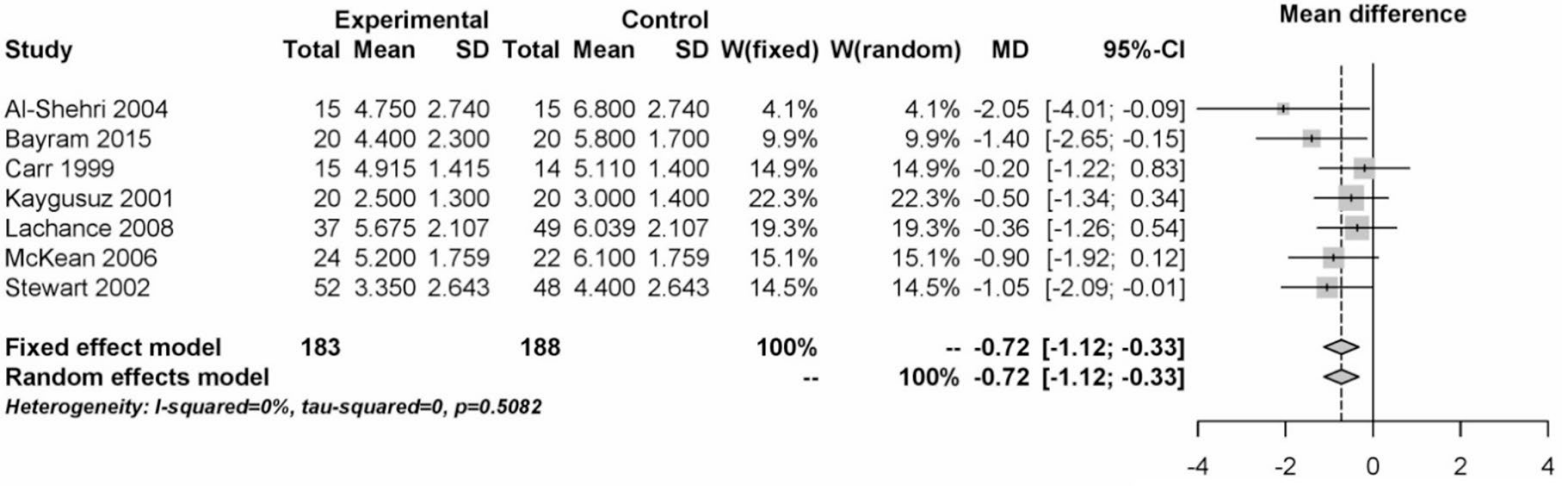
Forest plot for the meta-analysis of pain measurement on the third postoperative day for the use of steroids.

On the third postoperative day, 371 patients from 7 studies were analyzed. There was a benefit for the use of steroids compared to placebo. The pooled effect for both models was − 0.72 (95% CI: -1.12 - -0.33). There was no heterogeneity (I² = 0%, Fig. 14).

**Fig. 15:**
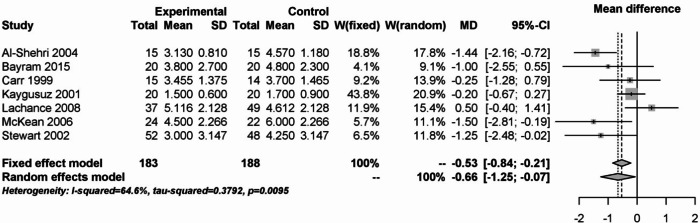
Forest plot for meta-analysis of pain measurement on day 7 postoperatively for the use of steroids.

The analysis of day 7 postoperatively is shown in Fig. 15. Steroids were able to significantly reduce pain with substantial heterogeneity (I² = 64.6%).

### Local anaesthetics

Local anesthetics are the largest drug class in this meta-analysis. A total of 35 references were analyzed^27,30,31,40–71^.

**Fig. 16:**
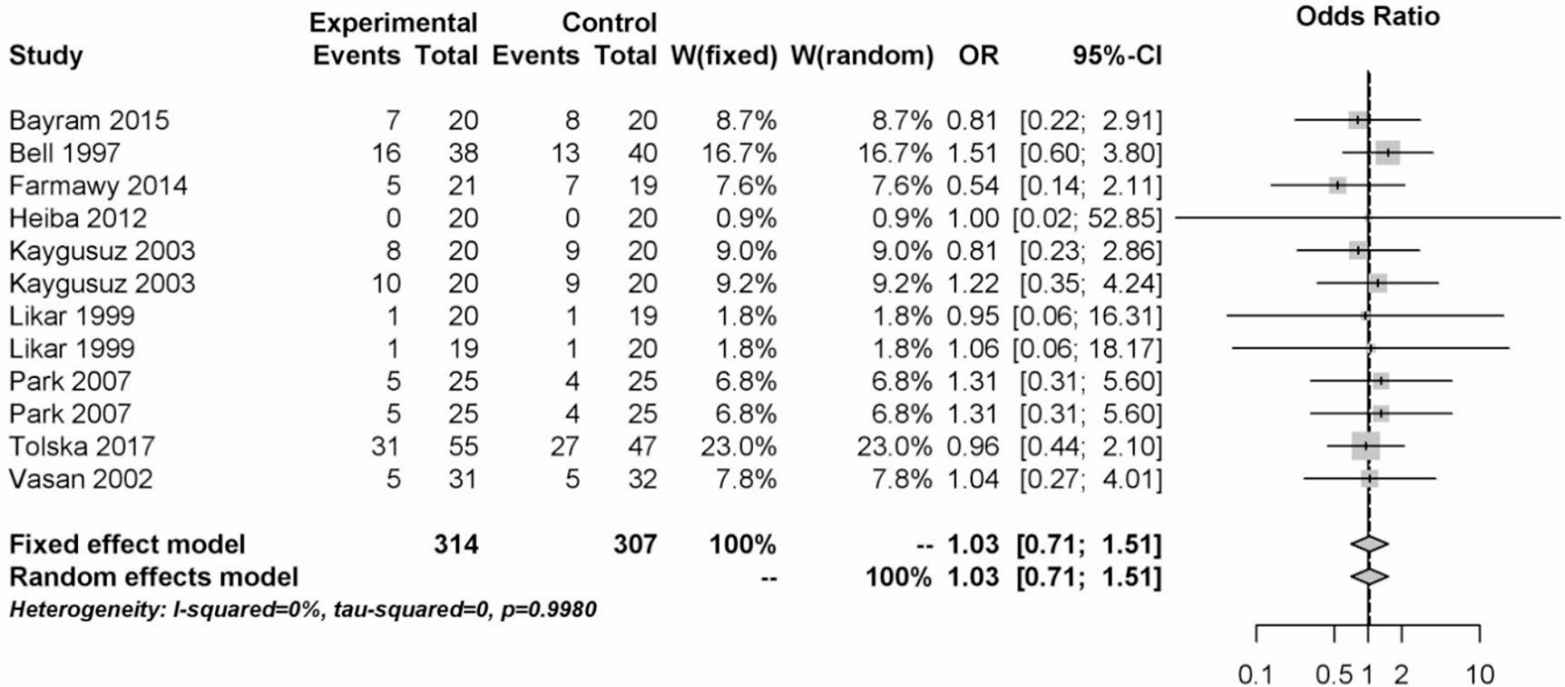
Forest plot for meta-analysis for postoperative nausea and vomiting for the use of local anaesthetics.

12 studies were used to analyze PONV among local anesthetics. This is shown in Fig. 16. In both models, there was no difference between the local anesthetic group and the placebo, opioid or corticosteroid control group (OR: 1.03; 95% CI: 0.71–1.51). The result was not heterogeneous (I² = 0%).

**Fig. 17:**
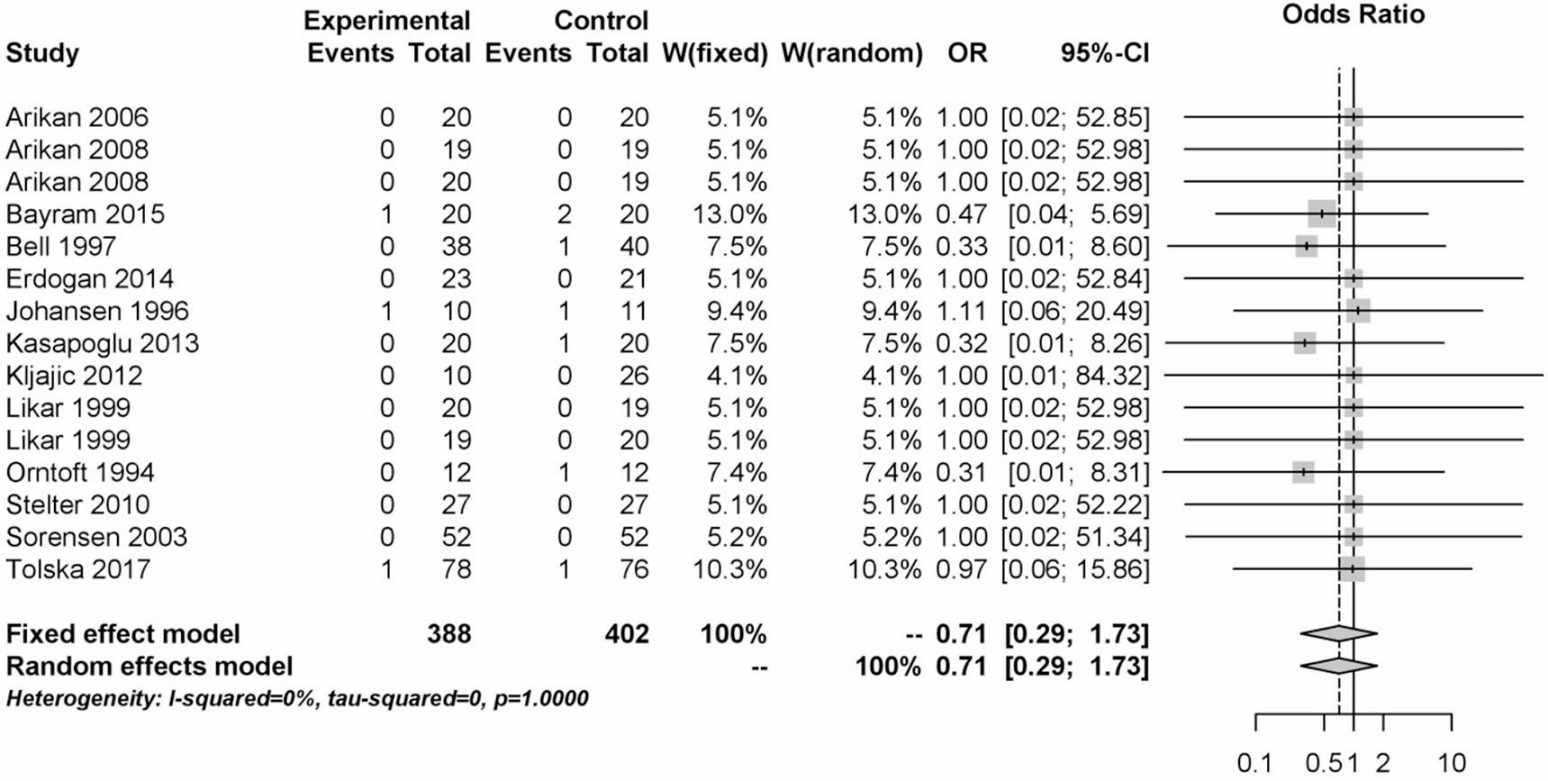
Forest plot for meta-analysis for primary postoperative bleeding for the use of local anaesthetics.

**Fig. 18:**
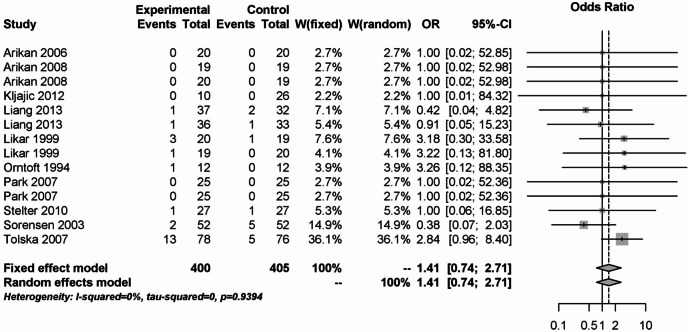
Forest plot for meta-analysis for secondary postoperative bleeding for the use of local anaesthetics.

In addition, local anesthetics had no significant effect on postoperative bleeding rates, either primary or secondary. This is shown in Figs. 17 and 18. 15 studies with 790 patients were included in the analysis of primary postoperative bleeding and 14 studies with 805 patients in the analysis of secondary postoperative bleeding. The OR was 0.71 (95% CI: 0.29–1.73) for primary postoperative bleeding and 1.41 (95% CI: 0.74–2.71) for secondary postoperative bleeding, respectively. There was no statistical heterogeneity in either analysis (I² = 0%).

**Fig. 19:**
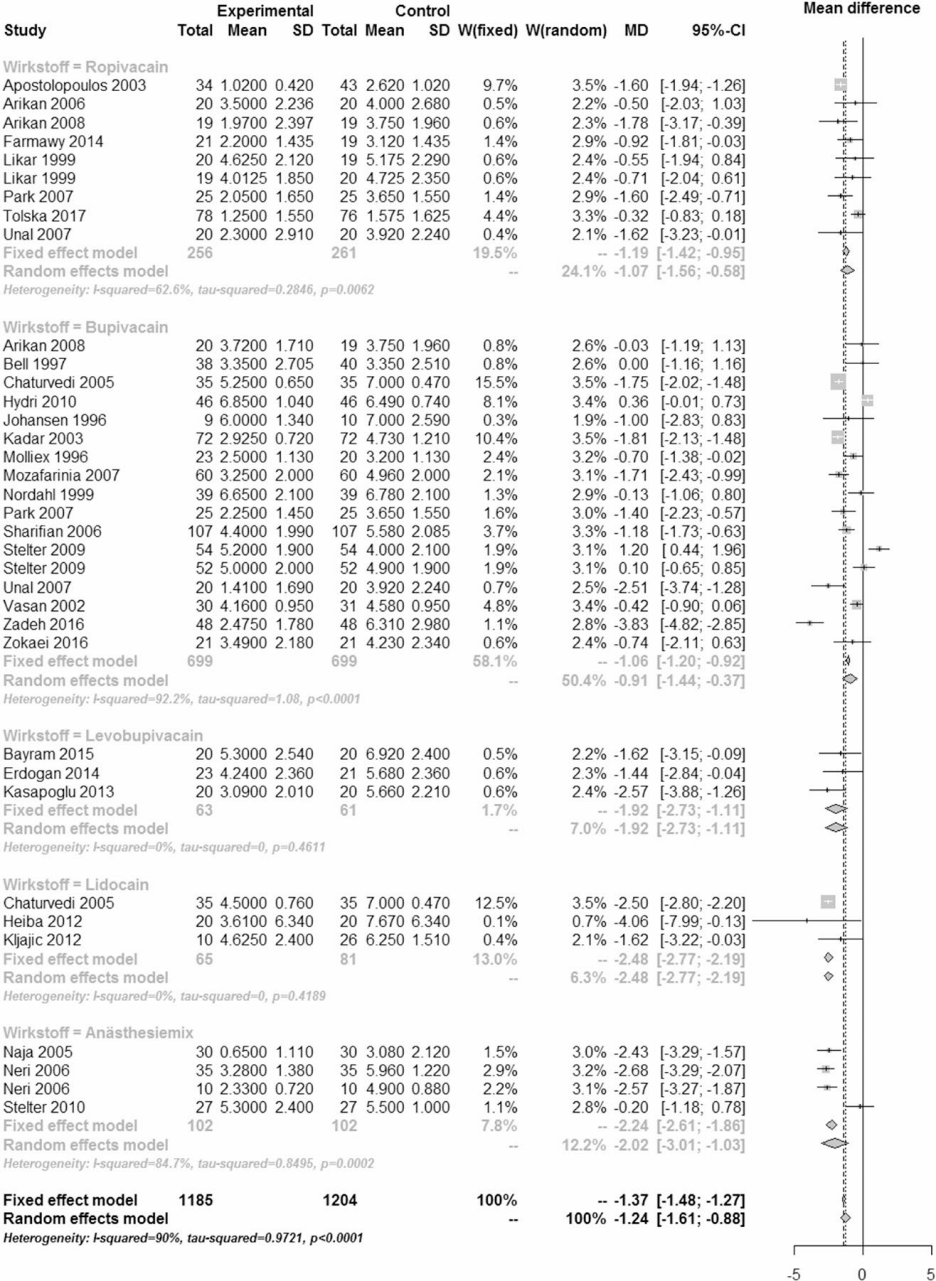
Forest plot for meta-analysis of pain measurement within the first 24 h for the use of local anaesthetics.

**Fig. 20:**
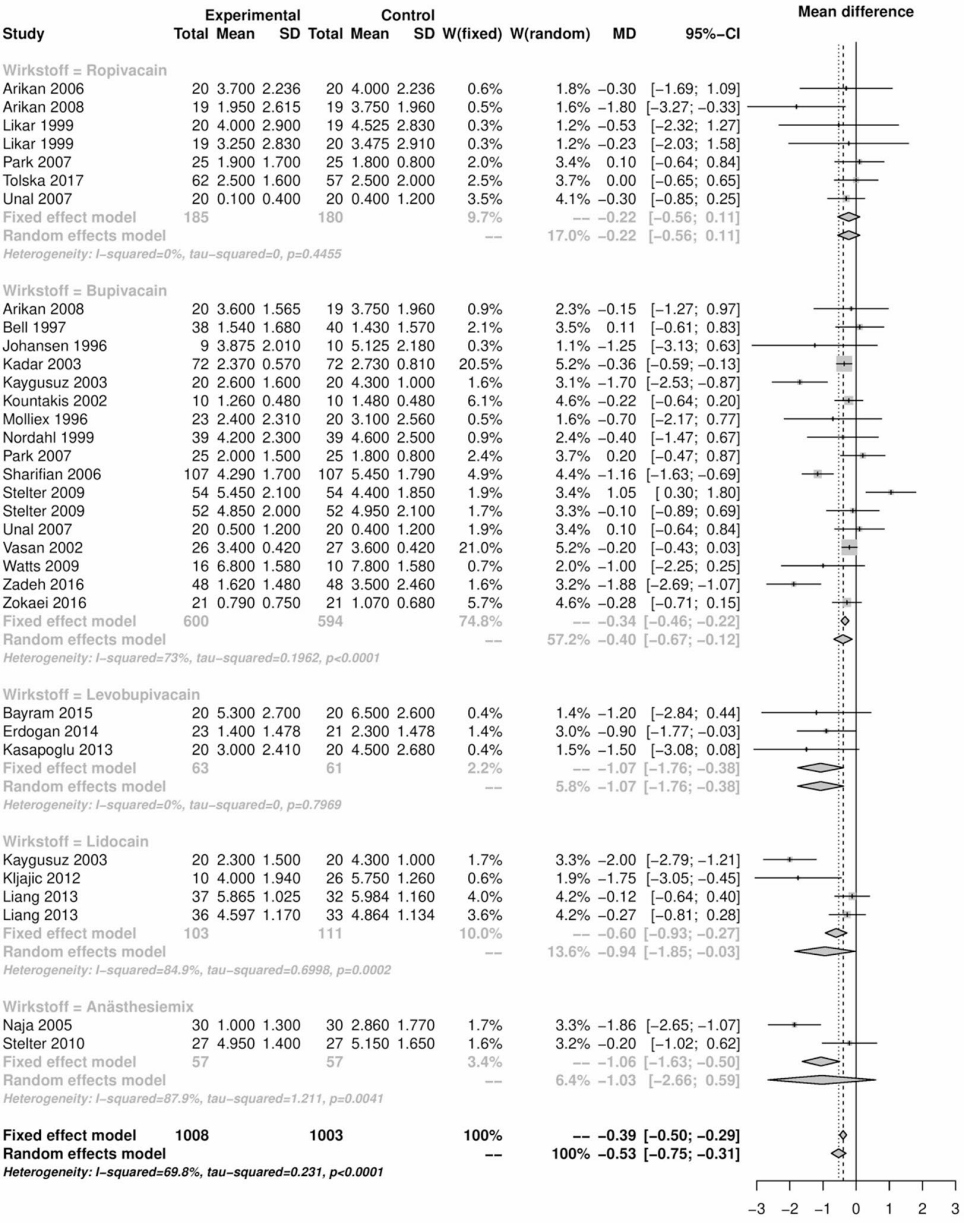
Forest plot for meta-analysis of pain measurement on the first postoperative day for the use of local anaesthetics.

**Fig. 21:**
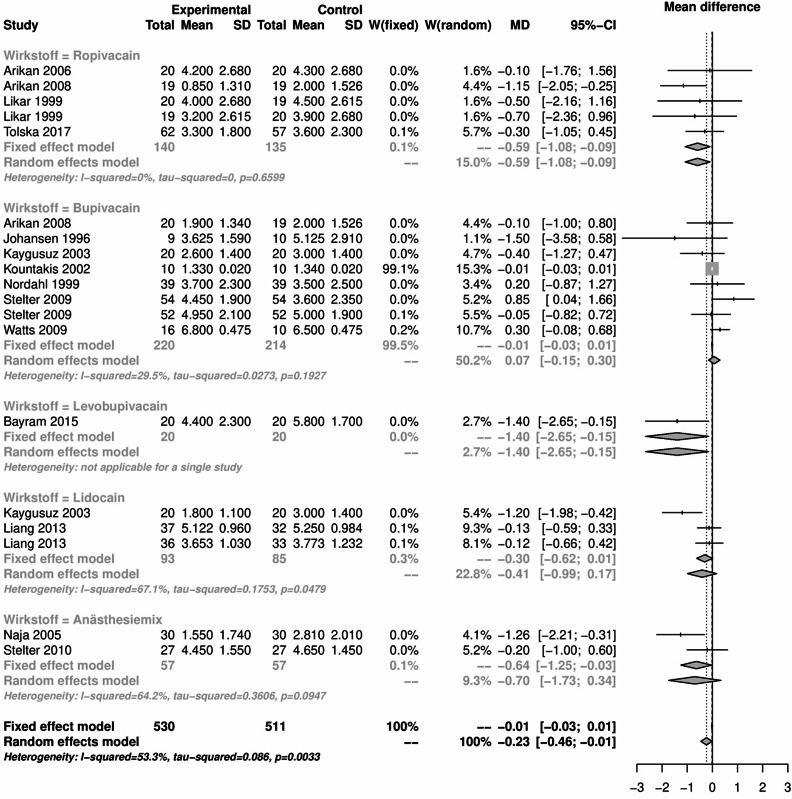
Forest plot for meta-analysis of pain measurement on the third postoperative day for the use of local anaesthetics.

**Fig. 22:**
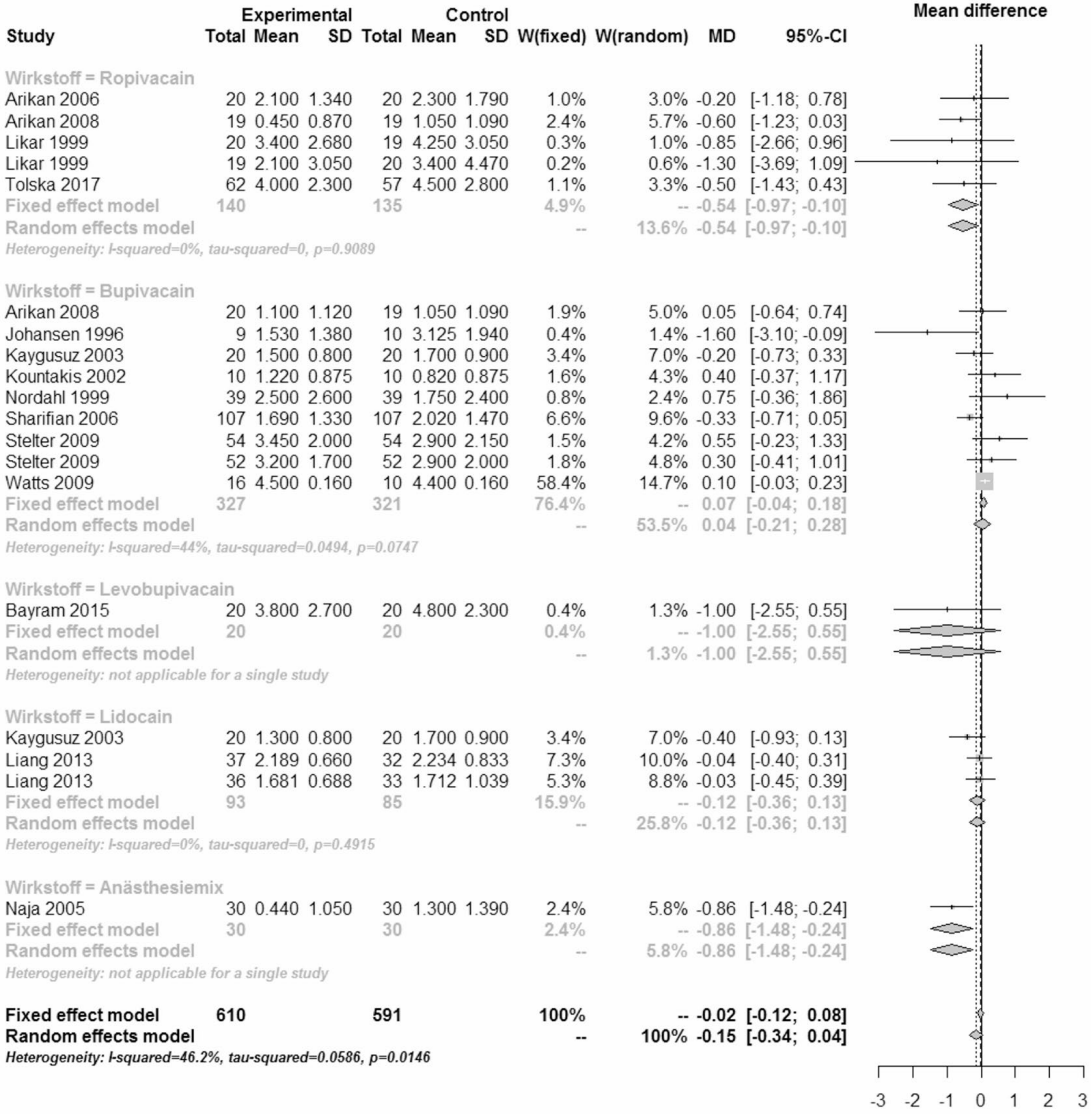
Forest plot for meta-analysis of pain measurement on the 7th postoperative day for the use of local anaesthetics.

The results of the pain analysis are shown in Figs. 19, 20, 21 and 22. In the first 24 h after tonsillectomy, 1185 subjects in the local anesthetics group and 1204 in the control group were analyzed. Local anesthetics were able to significantly reduce pain. The mean difference was − 1.37 (95% CI: -1.48 - -1.27) for the fixed effects model and − 1.24 (95% CI: -1.61 - -0.88) for the random effects model. However, the result was highly heterogeneous (I² = 90%).

The subgroup analysis of the individual agents showed that this heterogeneity was mainly due to the drug group bupivacaine (I² = 92.2%) and anesthesia mixture (I² = 84.7%). Despite their significant heterogeneity, the mean difference of the 1398 patients in the bupivacaine subgroup was − 1.06 (95% CI: -1.2 - -0.92) in the fixed effects model and − 0.91 (95% CI: -1.44 - -0.37) in the random effects model.

For the anaesthesia mix, the effect was even more pronounced in the 204 patients. The mean difference was − 2.24 (95% CI: -2.61 - -1.86) in the fixed effects model and − 2.02 (95% CI: -3.01 - -1.03) in the random effects model. Subgroup analysis of lidocaine and levobupivacaine showed significant pain reduction in homogeneous studies. The number of patients was small compared to the total number of patients (124 and 146 respectively), but the overall effect of -1.92 (95% CI: -2.73 - -1.11) in the levobupivacaine group and − 2.48 (95% CI: -2.77 - -2.19) in the lidocaine group indicated a significant reduction in pain.

## Discussion

The analyses of NSAIDs showed that the use of NSAIDs had no effect on the postoperative bleeding rate and PONV. The pain analysis of the first 24 h postoperatively showed that the administration of NSAIDs was able to significantly improve the pain. However, in the study by Louizos et al., the control group receiving rofecoxib was able to significantly reduce pain^13^. This publication also explains the considerable statistical heterogeneity that was present in this analysis. On postoperative day 1, the study situation was very heterogeneous. Only 4 of the 16 publications showed a significant benefit of NSAIDs^8,17,18,21^. The pooled effect was marginally significant in the fixed effects model, but not in the random effects model. NSAIDs were able to significantly reduce pain on postoperative day 3 and 7. However, there was considerable heterogeneity on postoperative day 7. This is due to the study by Courtney et al., in which the control group received tramadol^7^. Therefore, from the available analyses, it can be concluded that the use of NSAIDs is safe and can significantly reduce pain. The literature often refers the an increased risk of bleeding with NSAIDs. However, the present analysis did not show an increased risk of bleeding with the use of NSAIDs. Two meta-analyses from 2013 also found no increased risk of bleeding with NSAIDs^72,73^.

Steroids can not only significantly reduce pain, but also significantly reduce PONV. This was evident in the PONV analysis. At the same time, primary and secondary bleeding rates were not increased. Within the first 24 h and on day 3 after tonsillectomy, steroids significantly reduced patients’ pain. This positive effect was also observed on postoperative days 1 and 7. However, there was considerable heterogeneity in each case. Overall, steroids have a clear advantage. The safe use without increased bleeding rate and reduced incidence of PONV, as well as the significant pain reduction, support the use of steroids in the context of pain therapy in tonsillectomy. Several meta-analyses have already demonstrated this^74–79^. Intravenous steroids can significantly reduce PONV and postoperative pain.

The use of local anesthetics, which was the largest group of substances analyzed, had no effect on the rate of primary and secondary postoperative bleeding. There was also no difference in PONV compared to the control groups. Pain analyses showed a mixed picture. Although local anesthetics were able to significantly reduce pain within the first 24 h and on postoperative day 1, this was only possible in the presence of considerable heterogeneity. This heterogeneity was identified in the subgroup analysis and attributed to bupivacaine and anesthesia mix as well as lidocaine (only on postoperative day 1). No difference was found between the local anesthetics and the control groups on the postoperative days 3 and 7. In the first 24 h after tonsillectomy, lidocaine and levobupivacaine were able to significantly reduce pain without the presence of heterogeneity. Levobupivacaine was also able to do this on the 1st and 3rd days after surgery, but not on the 7th day. Something similar was found with ropivacaine. Ropivacaine was able to significantly reduce pain except on day 1, but sometimes only minimally or with significant heterogeneity. Overall, the use of local anesthetics for pain management after tonsillectomy requires further research. The variety of active ingredients and the many modes of application, such as preoperative or postoperative injections, impregnated swabs or sprays, make comparison difficult. Based on the available analyses, the use of local anesthetics can be considered safe in terms of secondary bleeding and PONV, but inadequate in terms of pain relief. This is also consistent with the data available to date. For example, while Grainger and Saravanappa recommend the use of topical local anesthetics in their meta-analysis^80^, Hollis et al. found no evidence^81^. Only levobupivacaine was able to significantly reduce pain without heterogeneity at the time points studied. The route of administration of levobupivacaine seems to play a minor role. Both topical application with swabs soaked in levobupivacaine^82^ and injection into the tonsil lodges^45^ had this significant effect on postoperative pain.

In conclusion, steroids and NSAIDs (alternatively coxibs) should be an integral part of pain management after tonsillectomy. A clear recommendation can be made for the intravenous administration of steroids. A single intraoperative dose of intravenous dexamethasone significantly improved both pain and the incidence of PONV. In addition, steroids do not affect the risk of postoperative bleeding. Among the local anesthetics, levobupivacaine was identified as a significant analgesic substance. However, the use of local anesthetics in the perioperative period requires further research. Although the local anesthetics used did not adversely affect the bleeding rate or PONV, there was no evidence of effective pain relief except for levobupivacaine. Therefore, only the preoperative injection of levobupivacaine into the tonsil lodges can be recommended.

The publication bias was minimized through the searches in the four databases mentioned and through the hand search. The study collective can be regarded as representative. A relevant selection bias cannot be identified. The studies were selected on the basis of strict criteria. In this study, RCTs were included throughout, as they show an extremely high level of evidence. Studies that performed additional operations in addition to tonsillectomy or used a pain scale other than the one mentioned were excluded. Furthermore, care was taken to ensure that the pain analysis covered both the early phase in the first few days postoperatively and the late phase, in particular the second peak of the bleeding complication.

One limitation of this study is that there was no separate investigation between studies on pain therapy in children and adults. In addition, there is the so-called language bias. Publications that were not published in German or English were not included.

## Methods

### Literature search

The Cochrane Library (https://www.cochranelibrary.com), Ovid Technologies (https://www.ovid.com), PubMed (https://pubmed.ncbi.nlm.nih.gov) and Web of Science (https://webofknowledge.com) were searched for literature on pain management after tonsillectomy between 1908 and 2019. The term “pain therapy after tonsillectomy” was used to search the databases and was supplemented by the different medications.

### Selection of cases

Randomized controlled trials (RCTs), systematic reviews and meta-analyses in German and English were included to evaluate pain management in children and adults of all ages after tonsillectomy. The different types of treatment were compared in comparison with the use of placebo, other treatments or no specific treatment. Studies were included if they documented pain using a visual analog scale (VAS), a verbal rating scale (VRS) or a numerical rating scale (NRS) were considered. Studies that did not meet these criteria were excluded from the meta-analysis. In the most cases, pain was assessed by the patients themselves or, in case of children, by their caregivers or parents. Studies with other or additional surgical procedures such as tonsillotomy, adenoidectomy or adenotonsillectomy and studies with unclear pain documentation or pain scales other than VAS, VRS or NRS were not included in the analysis.

### Data extraction

The primary endpoints were quantitative pain measurements at various time points during the first 24 h and on the postoperative day 1, 3 and 7. Postoperative bleeding, postoperative nausea with or without vomiting were selected as secondary endpoints.

### Statistical analyses

The results of meta-analysis are presented using forest plots. The forest plots show the results of fixed effects and random effects model, odds ratio (OR), mean difference (MD) and 95% confidence interval (CI). Publication bias was assessed using Egger’s test for funnel plot asymmetry. The I² statistic was used to quantify statistical heterogeneity.

## Data Availability

Data is provided within the manuscript. All authors had full access to all of the data in the study. KG and OGL take responsibility for the integrity of the data and the accuracy of the data analysis. KG and OGL should be contacted if someone wants to request the data from this study.
